# Systematic status of true katydids *Sathrophyllia* (Orthoptera, Tettigonioidea, Pseudophyllinae) from Pakistan, with description of two new species

**DOI:** 10.3897/zookeys.466.8423

**Published:** 2014-12-18

**Authors:** Riffat Sultana, Waheed Ali Panhwar, Muhammad Saeed Wagan, Imran Khatri

**Affiliations:** 1 Department of Zoology University of Sindh, Jamshoro, Sindh, Pakistan; 2 Department of Entomology, Sindh Agricultural University Tandojam, Sindh, Pakistan

**Keywords:** *Sathrophyllia*, new record, systematics, identification, new species, Pakistan

## Abstract

The genus *Sathrophyllia* Stål, 1874 from Pakistan is reviewed with four species recorded. The diagnostic characters are given and two new species *Sathrophyllia saeedi* **sp. n.** and *Sathrophyllia irshadi* **sp. n.** are described. In addition to that Sathrophyllia nr. rugosa (Linnaeus, 1758) and *Sathrophyllia femorata* (Fabricius, 1787) are re-described. Further information on the distribution and ecology of the species is given and a key to studied species of *Sathrophyllia* is presented. *Sathrophyllia femorata* (Fabricius, 1787) and *Sathrophyllia rugosa* (Linnaeus, 1758) are recorded from Rawalakot (KPK) and Tharparker (Sindh), Pakistan for first the time.

## Introduction

Bushcrickets or katydids belonging to the Tettigonioidea consume a wide variety of agricultural crops including forests, fruit orchards, and berry shrubs, and many species are ecologically associated with forest biocenoses, damaging trees and shrubs in addition to herbaceous plants (Riffat and Wagan 2012).

*Sathrophyllia* was erected by Stål in 1874 with type species *Sathrophyllia fuliginosa*. This genus consists of six species Barman (2003). Previously, many authors Stål 1874; Brunner 1893,^1895^; Kirby 1906; Karny 1923, 1924; Beier 1954, 1962, 1963; Otte 1997) carried out work on the morphology and taxonomic status of *Sathrophyllia* (true katydids) from tropical Asia and southern Arabia including the Indian subcontinent; after this there are no updated records available on this genus.

The present study fills some gaps in the existing knowledge. Furthermore, the addition of two new species proved to be a contribution to the biodiversity of *Sathrophyllia* fauna. Additionally, the findings of the present study will be useful in making predictions about the relationship between the species and for accurate identification in the future.

## Material and methods

The adult *Sathrophyllia* were collected from meadow grass, bushes, with mixed vegetation (herbs, shrubs and grasses) and tress with the help of traditional insect hand-nets (8.89 cm in width and 50.8 cm in length). For killing and preservation of specimens, the standard entomological methods described by Vickery and Kevan (1983) and Riffat and Wagan (2012) were adopted for all collected species. Identification of specimens was carried out under a stereoscopic dissecting binocular microscope (OLYMPUS SZX7, SZ2-ILST) with the help of keys and descriptions available in the scientific literature. The diagrams were all drawn with the help of an “Ocular Square Reticule” fitted in one eyepiece of the binocular microscope. All measurements are given in millimeters and were made with scales, dividers, and ocular square reticules. All the material is deposited in the Sindh Entomological Museum (SEM) Department of Zoology, University of Sindh, Jamshoro, Pakistan.

## Results and discussion

### Checklist of *Sathrophyllia* species

*Sathrophyllia arabica* Krauss, 1902 Arabian Peninsula

*Sathrophyllia cristata* Beier, 1954 Indo-China, Thailand

*Sathrophyllia femorata* (Fabricius, 1787) Pakistan **new record**

*Sathrophyllia fuliginosa* Stål, 1874 Indian Subcontinent, Nepal

*Sathrophyllia rugosa* (Linnaeus, 1758) Pakistan **new record**

*Sathrophyllia acutipennis* Beier, 1954 Malesia, Borneo

*Sathrophyllia saeedi***sp. n.**

*Sathrophyllia irshadi***sp. n.**

### Key to species of *Sathrophyllia* Stål, 1874 occurring in Pakistan

**Table:** 

1	Pronotum with one pointed tooth at anterior side, 6.3 mm in length (Fig. 1a, b)	**2**
–	Pronotum without pointed tooth at anterior side, 9 mm in length (Fig. 2a, h)	***femorata***
2	Ovipositor 10.3 mm long, with small tooth on its apex (Fig. 1e)	**nr. *rugosa***
–	Ovipositor 18 mm long, without small tooth on its apex (Fig. 4e)	3
3	Pronotum with tubercles, brown from dorsal side, 9.5 mm in length (Fig. 4c, d)	***irshadi* sp. n.**
–	Pronotum without tubercles, paler from dorsal side, 10 mm in length (Fig. 3a, b)	***saeedi* sp. n.**

**Figure 1.**
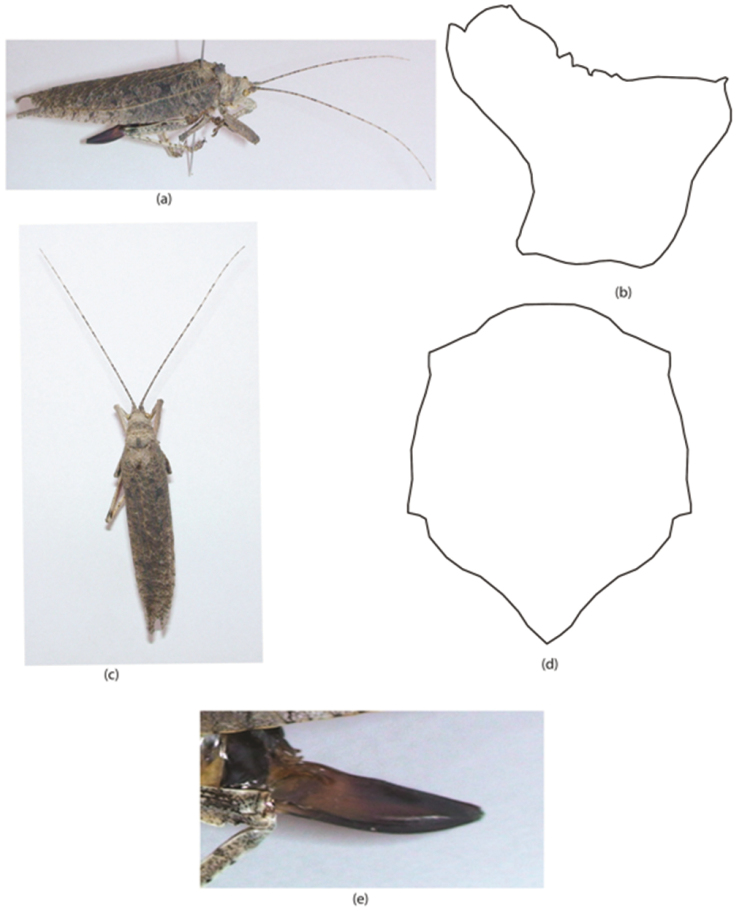
Sathrophyllia nr. rugosa Female; **a** adult LV **b** pronotum LV **c** adult DV **d** pronotum DV **e** ovipositor LV. Key: LV = lateral view, DV = dorsal view, VV = ventral view.

**Figure 2.**
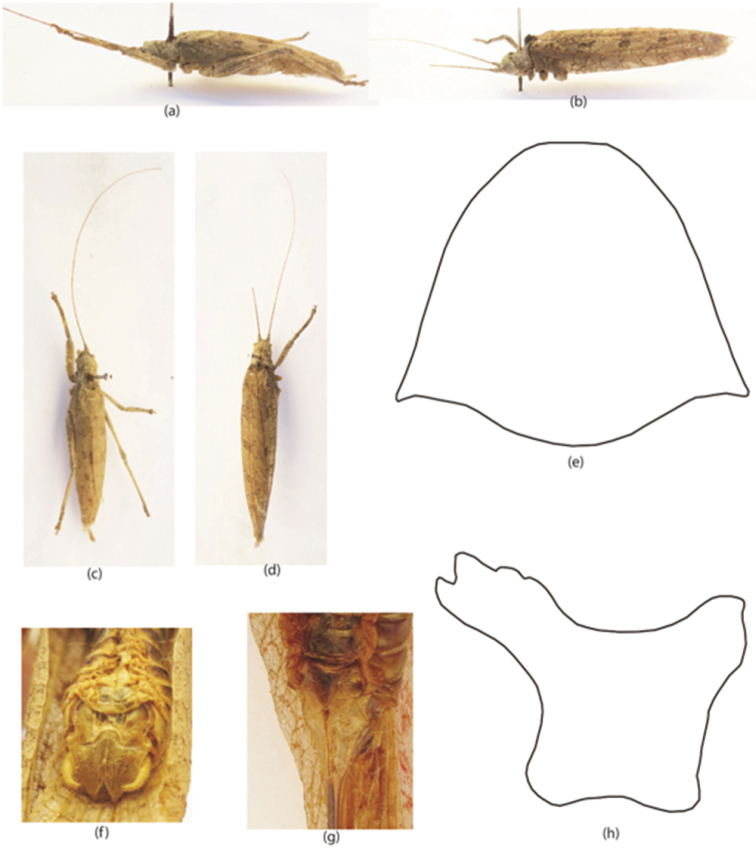
*Sathrophyllia femorata* **a–h** Male & Female **a** adult male LV **b** adult female LV **c** adult male DV **d** adult female DV **e** pronotum male DV **f** male subgenital fig **g** female subgenital fig **h** pronotum male LV.

**Figure 3.**
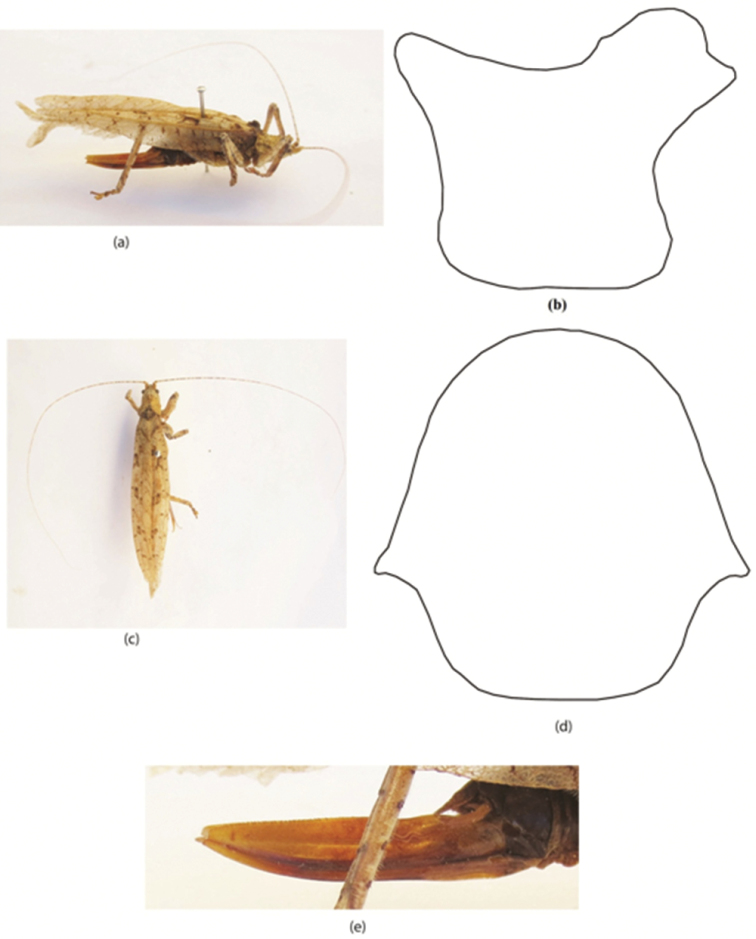
*Sathrophyllia saeedi* sp. n. female **a** adult LV **b** pronotum LV **c** adult DV **d** pronotum DV **e** ovipositor LV.

**Figure 4.**
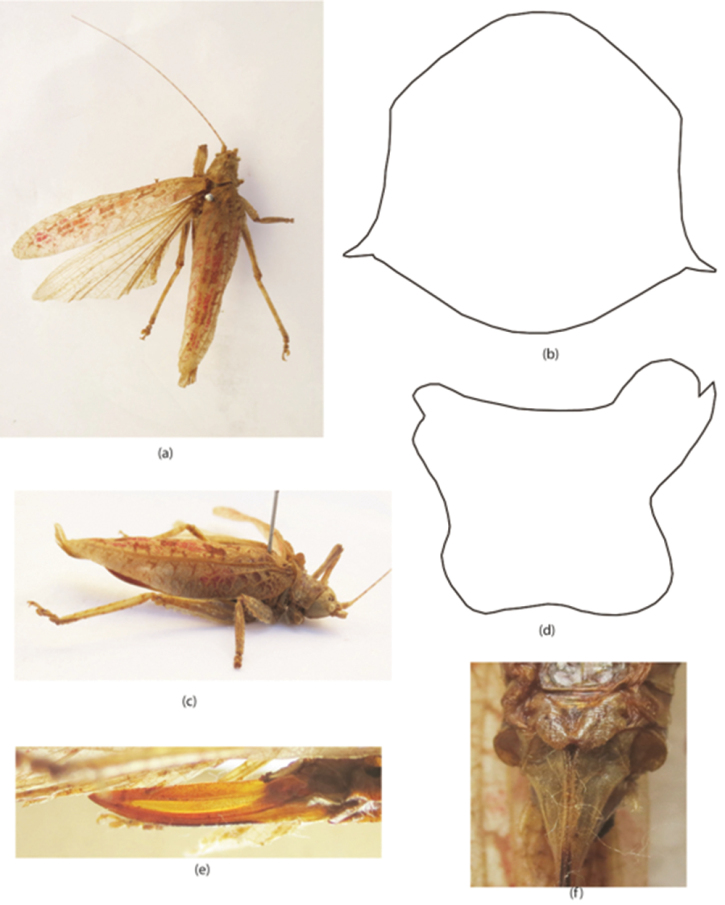
*Sathrophyllia irshadi* sp. n. female **a** adult DV **b** pronotum DV **c** adult LV **d** pronotum LV **e** ovipositor LV **f** subgenital fig VV.

### Tribe Cymatomerini

Taxon classificationAnimaliaOrthopteraTettigoniidae

Figs 1 , 2 , 3 , 4

#### Diagnosis.

Size medium to large, head usually short and rounded in appearance, face not slanting or flattened, head finely punctuated, forehead usually pale in color. Pronotum strong with or without tubercle on its apex. Ovipositor with or without spines on its apex.

### Sathrophyllia

Taxon classificationAnimaliaOrthopteraTettigoniidae

Stål, 1874

Dehaania Koningsberger, 1902.

#### Diagnosis.

Pronotum with or without tubercle on it, size medium or large (sometimes very large), head usually short and rounded, face not slanting or flattened; antennae longer than body, inserted between eyes. The fore margin of the tegmina is not or only very faintly undulate. In this character it differs from *Olcinia* Stal where the fore margin shows distinct lobes. In most species of *Sathrophyllia* the tegmina are tapering towards the apical part, not broadly rounded as in the genera *Tegra* Walker and in many species of the genus *Olcinia* Stal.

### Sathrophyllia nr. rugosa

Taxon classificationAnimaliaOrthopteraTettigoniidae

(Linnaeus, 1758)

Fig. 1a–e

#### Diagnostic features.

Dark brown tegmina with distinct dots (Fig. 1a, b, c). Pronotum has one pointed tooth on anterior side and several on posterior end; in the centre two transverse sulci are located just behind the middle (Fig. 1a–d). Centre of fore, mid, and hind femur with rounded dots on its lower side. Tegmina slightly shorter than wings. Ovipositor with small teeth on its apex (Fig. 1e).

#### Length measurements.

♀: pronotum, 6.3 mm; tegmina, 42 mm; femur, 10.3 mm; tibia, 8.7 mm; ovipositor, 10.3 mm; total body length, 25.9 mm.

#### Material examined.

Pakistan, Sindh, Tharparkar, Mithi, 1♀, 10.viii.2013, 24.7400°N, 69.8000°E (leg. Riffat S & Waheed AP).

#### Remarks.

This species is very closely related to *Sathrophyllia rugosa* (Linnaeus, 1758) but it differs due to following morphological analysis: it is smaller in size, the presence of serration on the ovipositor, coloration and width (4.2 mm). Furthermore, *Sathrophyllia rugosa* was described from Himalayas and the high altitudes and colder areas but the present specimen is coming from the desert area of Tharparkar, Sindh. Despite is uncertain placement, it is a new record for the area. The present investigation confirms the statement of the great Sir Uvarov (1924) that “The desert of Sindh harbours striking Orthoptera”. We agree on Sir Uvarov’s statement.

#### Ecology.

Sathrophyllia nr. rugosa has been collected from flat habitats. These habitats are usually surrounded by sand dunes comprising of sandy loam soils supporting a large number of taxa. The community formation of tree species such as *Prosopis cineraria* and *Tamarix aphylla* is present. Furthermore, katydids were also noted to have a close association with *Citrllus colycynthis*, *Dactylotenium scindicum* and *Poa tenella* in the survey areas.

### Sathrophyllia femorata

Taxon classificationAnimaliaOrthopteraTettigoniidae

(Fabricius, 1787)

Fig. 2a–h

Sathrophyllia orientalis (Rehn, 1909) 200: figs 22, 23.
Sathrophyllia punctifrons Karny, 1927, p. 8.

#### Diagnostic features.

Generally brown in color, suffused with drab at tegmina at the base of costal field and to a lesser degree on the distal half (Fig. 2a, b); nodes on the veins of the distal half of tegmina brown; eyes brown, antennae annulate with dark brown, median limbs irregularly spotted and caudal tibiae incompletely annulate with brown coloration; genicular part of caudal femora brown; abdomen blackish brown (Fig. 2c, d, e). Size medium, with a slightly depressed form; head smooth, depressed with dorsal length nearly two thirds of the pronotum. Pronotum strongly tuberculate (Fig. 2b, h); occiput slightly descending to very broad intraocular region. Fastigium of vertex sharply and considerably produced, moderately tapering, proximal width slightly exceeding half the width of the compound eyes; dorsum of fastigium deplanate, with a slight medial longitudinal sulcus. Lateral carina indicated by pair of rows of tubercles. Tegmina of peculiar texture, with low nodes of short cross-veins. Wings extended to tip of closed tegmina; mesosternum strongly transverse. Ovipositor without tooth on its apex. Cerci nearly reaching tip of subgenital fig, straight, robust, tapering at proximal two thirds. Subgenital fig produced into pair of depressed styliform processes of fig, slightly broader, apices bluntly acute (Fig. 2f–g).

#### Length measurements.

♂ pronotum, 9 mm; tegmina, 41 mm; femur, 16 mm; tibia, 15 mm; total body length, 30 mm. ♀, pronotum, 11 mm; tegmina, 46 mm; femur, 18 mm; tibia, 16 mm; ovipositor, 20 mm; total body length, 32 mm.

#### Material examined.

Rawalakot 1 ♂ & 1♀, 11.ix.2013, 33.51°N, 73.45°E (leg. Riffat S & Waheed AP).

#### Remarks.

Earlier, Brunner (1893) provided a revision of the genus *Sathrophyllia* from Italy and reported that *Sathrophyllia femorata* occurs in maximum numbers in Genova. At present we have reported 1♂ & 1 ♀ of this species from Pakistan, which are also new records for Rawalakot. The present study recommends that more detail surveys are needed to explore areas in order to improve the knowledge of this genus.

#### Ecology.

During the field surveys, it was observed that *Sathrophyllia femorata* fed exclusively in the cultivated field habitats occurring near valleys. These valleys were dominated by the grasses *Cynodon dactylon* and *Desmostachya*-*Brachiaria*. Present study suggests that most probably they feed on these grasses but our later study with more specimens will confirm this fact. Marini et al. (2010) also stated that as grasses having favorable of moisture level that attract species for their essential activities e.g breeding, feeding and overwintering. Possibly the grasses accumulate more visiting species to these habitats by providing appropriate environment for foraging and reproduction.

### Sathrophyllia saeedi sp. n.

Taxon classificationAnimaliaOrthopteraTettigoniidae

http://zoobank.org/9733C527-13B3-429F-ACB4-EB8C814367F3

Fig. 3a–e

#### Diagnosis.

This new species is closely related to *Sathrophyllia femorata* but differs in coloration and body size and in having a unique light brownish coloration. It is also smaller in size than *femorata* by approximately 3 mm. Body medium size; pronotum with tubercles and tegmina wider in the middle slightly tapering at apex. Ovipositor small in size, thick at its basal part and serrated at apex (Fig. 3e).

#### Description.

Head short, rounded, slightly ovoid at apex; pronotum with tubercles (Fig. 3b, d); size medium; antennae longer than the body length (Fig. 3a, c). Fastigium of frons with brownish spots. Pronotum paler in color dorsally, brown at posterior part. Eyes ovoid, brownish in color. Body light brown in color; antennae yellowish at scapus and with brownish spots; fastigium with brownish spots; abdomen brownish in color. Tegmina have brownish or dark brownish patches in a lunar shape. Ovipositor small, thick at its basal part, and serrated at apex (Fig. 3e). Ovipositor brown at base and over-all yellowish in color.

#### Length measurements.

♀: pronotum 10 mm, tegmina 47 mm, femur 19 mm, tibia 16 mm, ovipositor 18 mm, total body length 29 mm.

#### Material examined.

Rawalakot, 1♀, 11.ix.2013, 33.51°N, 73.45°E (leg. Riffat S & Waheed AP).

#### Remarks.

This new species is closely related to *Sathrophyllia femorata* but differ in the tegmina. The tegmina of *femorata* is of a peculiar texture with low nodes of short cross veins, whilst the tegmina in new species is brownish and has dark brownish patches on it producing a lunar shape.

#### Ecology.

*Sathrophyllia saeedi* apparently has been found in the agricultural/cultivated field habitats between dunes in valleys where *Desmostachya*-*Brachiaria cynodon* was dominant. Moreover, the valleys were comprised of different plant communities such as *Achyranthus aspera*, *Alhagi maurorum*, *Dactyloctenium aegypticum*, *Cenchrus ciliaris*, and *Cynodon dactylon*. The present study suggests that few grasshopper species were probably able to utilize these resources of such a habitat for promoting their breeding and feeding activities.

#### Derivatio nominis.

This new species is named in honor of Prof. Dr. Muhammed Saeed Wagan, a renowned taxonomist and ex-chairperson, Department of Zoology, University of Sindh, and the person who opened the door of entomology to us.

### Sathrophyllia irshadi sp. n.

Taxon classificationAnimaliaOrthopteraTettigoniidae

http://zoobank.org/60683DF8-542B-4249-893E-CF04E0496E63

Fig. 4a–f

#### Diagnosis.

This new species is closely related to *Sathrophyllia rugosa* but differs in coloration and body size: it has s brownish coloration and is also larger by 12.5 mm than *rugosa*. Furthermore, the tegmina of *Sathrophyllia irshadi* is of a peculiar texture with reddish patches on its surface. Body large in size; pronotum with numerous tubercles and tegmina well developed and much longer than the body length with reddish patches. Ovipositor wide, slightly tapering at apices thick at its basal part and but not serrated at apex (Fig. 4e).

#### Description.

Head rounded, ovoid at apices; pronotum with numerous tubercles (Fig. 4c, d); size large; antennae longer than the body length (Fig. 4a). Fastigium with brownish spots. Pronotum brown in color dorsally at anterior and posterior parts (Fig. 4a, b). Eyes ovoid, brownish in color. Body light brown in color; antennae yellowish at scapus with brownish spots; fastigium with brown spots; abdomen usually brown in color. Tegmina and wings fully developed; tegmina much longer than the total body length with reddish patches. Ovipositor wide, slightly tapers at apices thick at its basal part and but not serrated at apex (Fig. 4e, f). Ovipositor yellowish over-all and brownish at base.

#### Length measurements.

♀: pronotum 9.5 mm, tegmina 50 mm, femur 21.5 mm, tibia 16.5 mm, ovipositor 18 mm, total body length 38 mm.

#### Material examined.

Rawalakot, 1♀, 4.vi.2013, 33.51°N, 73.45°E (leg Riffat S & Waheed AP).

#### Remarks.

The new species is like that of *Sathrophyllia rugosa* but differ in body size and coloration. Furthermore, the specimen has been collected from Rawalakot which occurs above sea level whilst *Sathrophyllia rugosa* was collected from low altitude (24.7400° N, 69.8000° E) from Tharparker.

#### Ecology.

This species inhabits especially nutrient-rich grasslands. The surrounding plantations were covered by *Desmostachya*-*Brachiaria cynodon*, which were found to be dominant over this habitat. Moreover, the valleys were comprised of different plant communities i.e., *Achyranthus aspera*, *Alhagi maurorum*, *Dactyloctenium aegypticum*, *Cenchrus ciliaris* and *Cynodon dactylon*.

#### Derivatio nominis.

This new species is named in the honor of Muhammad Irshad Entomologist, NARC Islamabad, for his great contributions in the field of entomology.

## Supplementary Material

XML Treatment for Cymatomerini

XML Treatment for Sathrophyllia

XML Treatment for Sathrophyllia nr. rugosa

XML Treatment for Sathrophyllia femorata

XML Treatment for Sathrophyllia saeedi

XML Treatment for Sathrophyllia irshadi
